# Practical Human Anatomy

**Published:** 1886-10

**Authors:** 


PRACTICAL HUMAN ANATOMY. *A Working-guide for Students of Medicine, and a Ready Reference for Surgeons and Physicians. By* FANEUIL D. WEISSE, M.D. *Illustrated by 222 lettered plates, containing 321 figures; pp. vii, 456. New York:* WILLIAM WOOD & CO., *1886. Chicago:* W. T. KEENER.

The author has made a determined effort to overcome some of the chief difficulties surrounding the study of practical anatomy. That he has succeeded in a great measure, is disclosed by even a superficial examination of the work.

It is divided into twenty-seven dissections, each one of which consists of a descriptive text, followed by the plates necessary to its illustration. These latter are rendered especially valuable to the dissector by the plate references in the text. The student is thus enabled to verify his work by the plates, and to advance without confusion through the different parts of a dissection.

Twenty of the plates are selected from various works, and the remainder are reproductions of original drawings made from the author's own dissections. Too much praise cannot be given this part of the work, both for accuracy and high mechanical finish.

The publishers have issued the work in a creditable manner.

